# Migration of Regulatory T Cells to the Peritumor Microenvironment of Experimental Glioblastoma

**DOI:** 10.17691/stm2025.17.1.07

**Published:** 2025-02-28

**Authors:** E.P. Yanysheva, P.A. Melnikov, D.A. Chudakova, M.V. Shirmanova, V.P. Baklaushev, G.M. Yusubalieva

**Affiliations:** Junior Researcher, Laboratory of Solid Tumor Immunotherapy; Federal Center of Brain Research and Neurotechnologies of the Federal Medical Biological Agency of Russia, 1, Bldg. 10, Ostrovityanova St., Moscow, 117513, Russia; Junior Researcher, Laboratory of Cell Technologies; Federal Scientific and Clinical Center for Specialized Types of Medical Care and Medical Technologies of the Federal Medical Biological Agency of Russia, 28 Orekhovy Blvd., Moscow, 115682, Russia; PhD, Researcher, Laboratory of Neurobiology; Serbsky Federal Medical Research Centre of Psychiatry and Narcology, 23 Kropotkinsky Lane, Moscow, 119034, Russia; PhD, Senior Researcher, Laboratory of Neuroregeneration; Federal Center of Brain Research and Neurotechnologies of the Federal Medical Biological Agency of Russia, 1, Bldg. 10, Ostrovityanova St., Moscow, 117513, Russia; PhD, Deputy Director for Science, Research Institute of Experimental Oncology and Biomedical Technologies; Privolzhsky Research Medical University, 10/1 Minin and Pozharsky Square, Nizhny Novgorod, 603005, Russia; MD, DSc, Head of the Cell Therapy Development Department; Federal Center of Brain Research and Neurotechnologies of the Federal Medical Biological Agency of Russia, 1, Bldg. 10, Ostrovityanova St., Moscow, 117513, Russia; Head of the Biomedical Research Center; Federal Scientific and Clinical Center for Specialized Types of Medical Care and Medical Technologies of the Federal Medical Biological Agency of Russia, 28 Orekhovy Blvd., Moscow, 115682, Russia; Head of the Laboratory of Molecular Regeneration Mechanisms; Engelhardt Institute of Molecular Biology of the Russian Academy of Sciences, 32 Vavilov St., Moscow, 119991, Russia; Head of the Regenerative Medicine Laboratory; Pulmonology Scientific Research Institute of the Federal Medical Biological Agency of Russia, 28 Orekhovy Blvd., 115682, Russia; MD, PhD, Head of the Laboratory of Solid Tumor Immunotherapy; Federal Center of Brain Research and Neurotechnologies of the Federal Medical Biological Agency of Russia, 1, Bldg. 10, Ostrovityanova St., Moscow, 117513, Russia; Senior Researcher, Laboratory of Cell Technologies; Federal Scientific and Clinical Center for Specialized Types of Medical Care and Medical Technologies of the Federal Medical Biological Agency of Russia, 28 Orekhovy Blvd., Moscow, 115682, Russia; Senior Researcher, Laboratory of Molecular Regeneration Mechanisms; Engelhardt Institute of Molecular Biology of the Russian Academy of Sciences, 32 Vavilov St., Moscow, 119991, Russia

**Keywords:** regulatory T lymphocytes, tumor microenvironment, glioblastoma, glioma, migration, tumor

## Abstract

Glioblastoma is the most aggressive primary brain tumor with poor prognosis characterized by resistance to standard treatments and immune evasion. Regulatory T lymphocytes (Tregs) play a key role in immune suppression in the tumor microenvironment and can be used as targets for malignant gliomas therapy.

**The aim of the investigation** is to study migration of Tregs to the tumor site in the process of dynamic glioblastoma growth on the transgenic C57Bl/6-FoxP3-eGFP mouse line.

**Materials and Methods:**

The study was performed using the C57Bl/6-FoxP3-eGFP mouse strain, which allows for the detection of FoxP3-positive Tregs by fluorescent signal. Orthotopic glioblastomas were implanted by stereotactic injection of fluorescently labeled GL-261-BFP and GL-261-mScarlet tumor cell lines. Intravital confocal microscopy was used to monitor infiltration of the tumor site by immune cells, visualized by intravenous injection of fluorescently labeled antibodies against CD45. The results of intravital microscopy were confirmed by histological and immunohistochemical examination on days 3, 6, 9, 14, and 16 after the implantation. To assess the immunological status, tumor-infiltrating lymphocytes (TILs) were isolated from the brain and Tregs were counted using a flow cytometer (immediately after isolation and after cultivation for 2 weeks).

**Results:**

Intravital microscopy and brain slice studies have demonstrated infiltration of the glioblastoma site by Tregs, with the proportion of Tregs increasing with tumor progression (the increase in the absolute number of Treg was proportional to the increase in the number of glioma cells). Subsequent co-cultivation of isolated TILs with glioma cells revealed increase of Treg population within 2 weeks from 2.8% to >40%, confirming the activating effect of glioblastoma with respect to Tregs.

**Conclusion:**

The dynamics of GL-261 glioma microenvironment infiltration by Tregs has been investigated. The glioblastoma cells were shown to activate Tregs in the peritumor space *in vivo* and to promote their selective expansion when co-cultured with TILs *in vitro*. These data can be used for further studies on C57Bl/6-FoxP3-eGFP mice to find approaches to inactivate Tregs in glioblastoma.

## Introduction

Glioblastoma is the most common and almost incurable brain tumor. Currently, a five-year survival rate for patients with glioblastomas does not exceed 5–10%, according to different data sources [1]. This indicates the ineffectiveness of standard treatment modalities (maximum possible surgical resection, radiation therapy and chemotherapy with temozolomide and bevacizumab), therefore, there is an urgent need to develop new therapeutic approaches [2, 3].

Neo-adjuvant immunotherapy may be one of the possible solutions to this problem [4]. However, the first attempts of cell-based glioblastoma immunotherapy were not successful, largely due to its microenvironment, which evokes a high level of immunosuppression in the tumor site [5]. The rapid progression of glioblastoma often leads to the fact that patients seek specialized care when the tumor has a significant size and an immunosuppressive microenvironment, characterized by a decrease in CD4^+^ T helper cells, cytotoxic activity, and proliferation of CD8^+^ T lymphocytes as well as increased levels of Tregs and other non-tumor cells in the microenvironment [6, 7].

The tumor microenvironment is a complex system composed of many interacting components: tumor cells, including glioma stem-like cells, affect extracellular matrix, endotheliocytes, fibroblasts, mesenchymal stromal cells, and immune cells, contributing to the acquisition of a pro-tumor cell phenotype [8–10]. Regulatory T cells (Tregs), which under normal conditions play a role of suppressors of inflammatory and autoimmune reactions in the body, make the greatest contribution to the suppression of the natural antitumor immune response [11]. Thus, inactivation of Tregs is the most obvious problem in the development of targeted immunotherapy for glioma [12–15].

The study of Treg behavior during formation of the glioblastoma tumor microenvironment is an urgent task, which cannot be accomplished using the xenograft model of human glioblastoma in immunodeficient mice due to the defect in cell-mediated immunity in these animals. The solution of this problem lies in the possibility to use transgenic C57Bl/6-FoxP3-eGFP mouse strain, allowing for the detection of activated Treg in tissues. The sequence of enhanced green fluorescent protein (eGFP) was cloned into the first exon of transcription factor FoxP3 in this mice line. Activation of Tregs is always accompanied by FoxP3 expression [16], and in C57Bl/6-FoxP3-eGFP mice eGFP is co-expressed with FoxP3 in the same cells. This allows visualization of active regulatory CD4^+^ CD25^+^ FoxP3^+^ T cells using fluorescence microscopy in the appropriate channel (Ex — 475nm, Em — 508 nm). Treg detection in these mice is possible both on slices of the organ of interest and by real-time intravital confocal microscopy.

**The aim of our study** is to assess migration of regulatory T lymphocytes into glioma focus in the process of dynamic glioblastoma growth on the transgenic C57Bl/6-FoxP3-eGFP mice line.

## Materials and Methods

### Cell lines

In this work, the GL-261-mScarlet mouse glioma cell lines (transduced with red fluorophore protein mScarlet) and GL-261-BFP (tranceduced with blue fluorescent BFP), provided kindly by Alexey Stepanenko (Department of Neurobiology, Serbsky Federal Medical Research Centre of Psychiatry and Narcology, Moscow, Russia), were used to model experimental orthotopic glioblastoma.

After the removal from the cryobank, the cells were cultured in DMEM F12 medium supplemented with 20% fetal bovin serum (FBS) and 1% antimycotic/antibiotic; the culture confluency was assessed using Primo Vert light microscope (Zeiss, Germany). The cells were counted in the Countess 3 Automated Cell Counter (Thermo Fisher Scientific, USA).

Immediately before implantation into mice, glioma cells were detached from the culture dish using TrypLE enzyme. The harvested cells were washed from the medium with TrypLE, and re-suspended in sterile Dulbecco’s phosphate-salt buffer (DPBS) solution for subsequent injection. Tumor-infiltrating lymphocytes (TILs) from the glioblastoma tissue were obtained from 4 mice on day 6 after implantation (immediately after intravital microscopy) in compliance with the protocol described previously [16]. To co-cultivate glioma with TILs in flat-bottom adhesion plates, IMDM medium supplemented with 20% FBS and 1% penicillin/ streptomycin was used.

### Experimental transgenic mouse line

Syngeneic glioblastoma was modeled in 16 C57Bl/6-FoxP3-eGFP transgenic mice (animal facility of the Research Institute of Experimental Oncology and Biomedical Technologies, Privolzhsky Research Medical University, Nizhny Novgorod, Russia). The sequence of eGFP was cloned into the first exon of *FoxP3* gene of this mouse line to identify regulatory FoxP3^+^ CD4^+^ T cells.

All study protocols were approved by the local ethical committee of Federal Scientific and Clinical Center of the Federal Medical Biological Agency of Russia (Moscow, Russia), protocol No.7 of September 6, 2022, and were conducted in full compliance with the guidelines of work with laboratory animals (Recommendation of the Eurasian Economic Commission’s Board of November 14, 2023, No.33 “Guideline on Handling Laboratory Animals in Non- Clinical Trials/Studies”).

### Modelling of experimental glioblastoma

To model orthotopic syngeneic glioma, 4–5-week-old mice were anesthetized with Zoletil (40 mg/kg, 50 μl) and Romethar (8 mg/kg, 50 μl) and fixed through the ear canals in a stereotaxic system (RWD Life Science, China). Mice were scalped and a trepanation hole was made in the skull in the projection of the right cerebral hemisphere striatum using Drill Bits HM1005 0.5 mm, Round Tip (RWD Life Science, China). GL-261 cells (150,000 cells in 5 μl of sterile Dulbecco’s phosphatesalt buffer) were injected using a micropump at a rate of 1 μl/min. For immunohistochemical analysis and flow cytometry, the suspension of glioma GL-261- BFP cells was stereotactically injected into the brain striatum of 8 mice (of them 3 animals were perfused on day 9 and 3 animals on day 16). For intravital confocal microscopy, 5 μl of the GL-261-mScarlet glioma cell suspension were injected into the cortex of the right cerebral hemisphere of 8 mice (6 animals underwent lifetime microscopy on days 3, 6, and 9, and tumors were isolated in 2 remaining animals on day 6, which were used to obtain TILs for co-culturing with GL-261- mScarlet cells).

### Magnetic resonance imaging

Experimental glioblastoma was dynamically monitored in 5 of 8 mice with GL-261-BFP glioma on days 7, 14, 21 and in all mice with GL-261-mScarlet glioma on days 1, 9, and 16 after implantation before microscopy. The study was performed using the ClinScan 7T MRI scanner (Bruker Biospin, Germany) for small laboratory animals installed in the Center for Collective Use “Medical and Biotechnological Nanotechnologies” at Pirogov Russian National Research Medical University (Moscow, Russia). Scanning was performed under inhalation anesthesia with 1.5% isoflurane in an oxygen mixture at a flow rate of 250 ml/min using an EZ-7000 Classic System apparatus (E-Z Systems Inc., USA). T2 weighted images in axial, coronal, and sagittal projections were obtained using a brain coil for small laboratory animals.

### Intravital confocal microscopy

Intravital microscopy (IVM) was performed using Nikon A1 MP confocal microscope (Nikon, Japan) with a heated adapter stage optimized for the work with round coverslips 50 mm in diameter. Mice with superficial location of GL-261-mScarlet glioma in the cerebral cortex, confirmed by MRI, were anaesthetized with Rometar (8 mg/ml, 50 μl) and Zoletil (19 mg/kg); an intracranial window was formed under sterile conditions, then the animals were placed on the table so that the area of interest fitted tightly to the surface of the coverslip and was available for microscopy. To assess the overall infiltration of the tumor microenvironment by immune cells, 2 μl of the labeled Brilliant Violet 421 anti-mouse CD45 antibody solution (BioLegend, USA) in 100 μl DPBS was injected into the tail vein 15 min before IVM. The localization of labeled cells relative to the tumor focus was determined using stepwise imaging with shifting along the Z axis. Several tumor regions were scanned alternately for 120 min (1 frame 512×512 or 1024×1024 px every 40–60 s). Next, all the obtained images were combined into a “stack”, thus generating a 3D image of the tissue. Tregs were detected by eGFP signal; glioma cells were detected by mScarlet signal. Confocal microscopic images were processed using NIS Elements AR software (Nikon, Japan). After the completion of IVM, the intracranial window was covered with a skin flap, mattress suture was applied and treated with antiseptic solution.

### Immunofluorescence analysis on brain slices

Brain slices were examined in 6 mice with GL-261-BFP glioma implanted in the striatum on day 9 and 16 after implantation. Animals were deeply anaesthetized with an extremely high dose of propofol and transcardial perfusion with 4% paraformaldehyde solution was performed. After perfusion, the animals were decapitated and the brain was isolated using scissors and forceps. The isolated mouse brain was fixed for 48 h at 4°C in 4% paraformaldehyde solution and then 50 μm slices were prepared using the Microm HM 650V 5100mz vibratome (Campden Instruments, England).

### Immunocytochemical analysis

To identify Tregs in the glioblastoma microenvironment, the obtained slices were mounted on the glass for subsequent confocal microscopy. Before mounting, some slices were additionally stained with primary labeled antibodies to CD105 to visualize mesenchymal stromal cells. The stained slices were mounted on a slide using hypofluorescent Faramount Mounting Medium. Some slices were additionally stained with DAPI (blue fluorescent dye that binds to DNA) before mounting to contrast cell nuclei (1 μl per 100 μl buffer), and the confocal laser scanning microscopy was performed using Nikon Eclipse Ti-2 microscope (Japan). All obtained data were analysed using NIS Elements Viewer software v. 5.21.

### Isolation and flow cytometry of Tregs in tumorinfiltrating lymphocytes

To isolate TILs from intracranial tumor, animals were deeply anesthetized and cervical dislocation was performed. The skin and fur of the mice were treated with alcohol. After decapitation, small surgical scissors were used to open the scalp above the parietal bones, trim the edge of the skull, and carefully pull the bones apart. The mouse brain was extracted with forceps and placed in 15 ml falcon with DPBS + antibiotic-antimycotic. Subsequently, the olfactory bulbs and cerebellum were discarded in a Petri dish and the right and left hemispheres were separated from each other for later comparison (glioma was implanted in the right hemisphere, left hemisphere served as a control). Each hemisphere was separately crushed with the addition of DPBS to a slurry and passed twice through a cell filter with a 100 μm pore size. The resulting cells were centrifuged for 5 min (400 g, 3 accelerations, 3 decelerations), and the precipitate was suspended in buffer solution for flow cytometry (Milteni Biotech, Germany). The remainder of the TILs isolated from the brain were cultured and expanded as previously described [17].

The proportion of CD4^+^ CD25^+^ FoxP3^+^ T cells in the obtained TILs was determined. The study was performed using MACSQuant Analyzer 16 flow cytometer (Miltenyi Biotec, Germany) immediately after isolation of TILs on day 6 of the tumor development and during subsequent cultivation of the cells for 14 days. The results were analysed using FlowJo v. 9 software.

## Results

### MRI characterization of intracranial GL-261 glioblastoma growth

**Figure 1.**
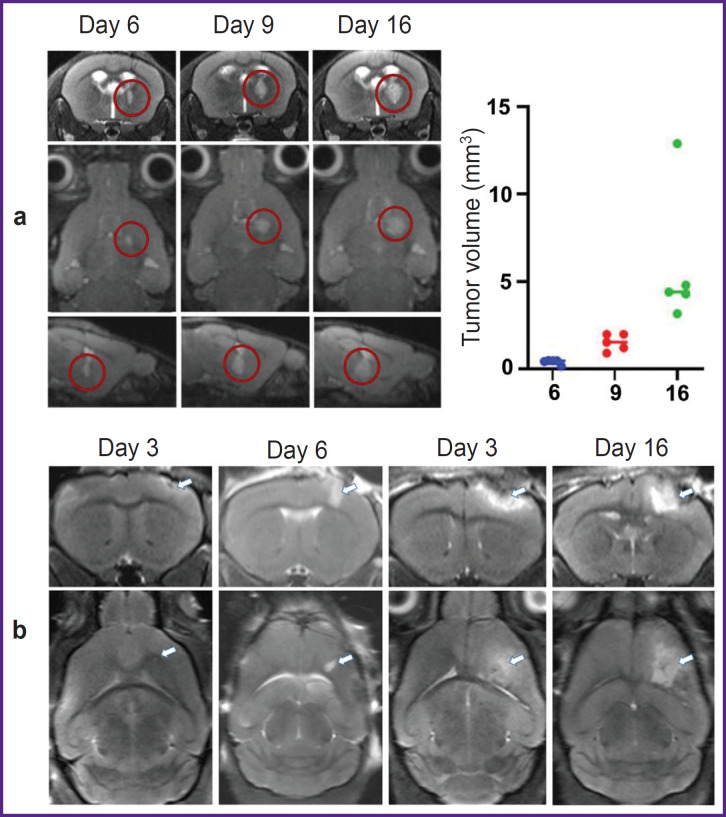
MRI data on the dynamics of GL-261- BFP glioma growth: (a) GL-261-BFP cells were inoculated into the right striatum of the mouse brain; MRI was performed on days 6, 9, and 16 after tumor implantation; representative images are shown; the tumor site is circled in red; right: dynamics of the implanted tumor volume determined by the ellipse volume formula; (b) MRI imaging of the cortical tumor for intravital microscopy on days 3, 6, 9, and 16; arrows show the tumor focus

For initial characterization of glioma growth, dynamic MRI was performed in 5 animals on days 6, 9, and 16. Glioma was detected on day 9 in all animals and by day 16 its volume reached 5.5 [3.8; 13.4] mm^3^ (Figure 1 (a)). For cortical localization of glioma, it was necessary to choose the time frame most suitable for intravital studies (with the most superficial location of the tumor). For this purpose, MRI studies were performed daily after the implantation of GL-261- BFP in the cortex. The tumor growth became detectable on days 3–4 (Figure 1 (b)). The tumor size gradually increased up to day 6, the superficial localization of the glioma was preserved. On day 9 and especially on day 16, a significant increase in the tumor volume and its spreading deep into the brain was observed. Based on the character of glioma growth according to MRI data, the time points of 3, 6, 9, and 16 days were chosen for intravital microscopy and immunofluorescence studies.

### Intravital confocal microscopy

**Figure 2.**
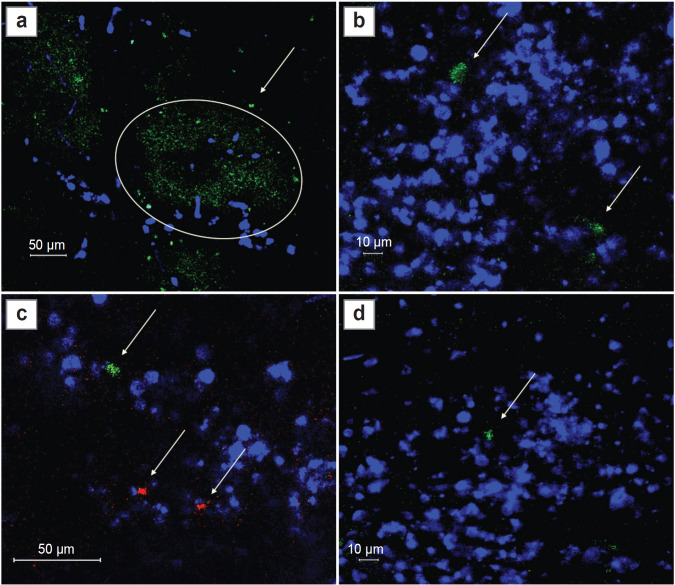
Intravital confocal microscopy from the cerebral cortex surface through the intracranial window on day 3 after implantation: (a) eGFP signal (green channel, shown by ellipse) in the zone the needle track; (b) infiltration by CD45^+^ cells (antibody labeled with Brilliant Violet 421, blue channel) in the area of the sham track; (a)–(d) pool of leukocytes CD45^+^ and eGFP^+/^FoxP3^+^ Treg (green channel, shown by arrows) in the GL- 261-mScarlet implantation area (red channel, shown by arrows)

IVM was used to evaluate Treg infiltration in the process of intracranial glioma growth starting from day 3 after implantation of GL-261-mScarlet cells into the cortex. At this time point, the tumor size was such that the MRI pattern was almost indistinguishable from the MRI track in sham glioma implantation (see Figure 1 (b)). At this stage, IVM showed marked infiltration with CD45^+^ leukocytes starting from the soft dura in the zone of the needle track (Figure 2 (a)). Among all CD45^+^ cells, we observed only single FoxP3^+^ Tregs, detected in the mice by the eGFP^+^ signal, whose proportion did not exceed 1% (Figure 2 (b)–(d)).

**Figure 3.**
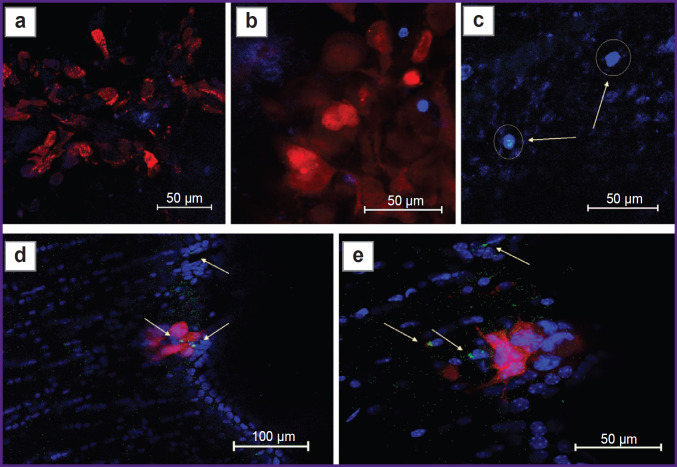
Intravital confocal microscopy from the cerebral cortex surface through the intracranial window on day 6: (a), (b) aligned images; (c) co-localization of signal from eGFP^+^/FoxP3^+^ and surface marker CD45; (d), (e) glioma cell invasion area surrounded by CD45^+^ immune cells. The proportion of eGFP^+^ cells is 5%. Red channel — signal from mScarlet (tumor cells); green channel — eGFP (FoxP3^+^ Tregs, shown by arrows), blue channel — pool of CD45^+^ leukocytes

On day 6 of tumor growth, there were more FoxP3^+^ Tregs eGFP^+^ signals in the tumor and peritumor space according to IVM data, but their share still did not exceed 5% of the total number of immune cells infiltrating the peritumor space (Figure 3).

**Figure 4.**
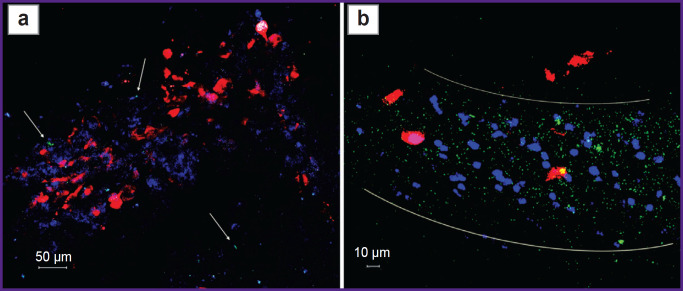
Intravital confocal microscopy of the glioma and periglioma zone through the intracranial window on day 9. Representative images: (a) localization of GL-261-mScarlet glioma in the right hemisphere cortex. FoxP3^+^ Tregs (green channel, shown by arrows) in the GL- 261-mScarlet glioma focus (red channel); (b) CD45^+^ immune cells (blue channel)

On day 9, we detected the maximum number of eGFP^+^, expressing FoxP3^+^ Tregs in the periglioma space. The number of Tregs in this period in the IVMdetected part of the tumor reached 10%, and in the peritumor space about 40% (Figure 4). At the same time, on day 9, only the glioma pole could be visualized by IVM. The bulk of the tumor was inaccessible for examination due to invasive growth beyond the intracranial window and deep into the brain. In order to visualize the entire tumor, an additional group of 6 C57Bl/6-FoxP3-eGFP mice with GL-261-BFP implantation in the striatum was used. Three animals were perfused on day 9 and the remaining three on day 16. Fluorescence imaging was performed on freshly prepared vibratome tumor sections. CD105^+^ tumor-associated stromal cells, which also play an important role in the immunosuppressive tumor microenvironment, were visualized in the red channel in this series of experiments.

**Figure 5.**
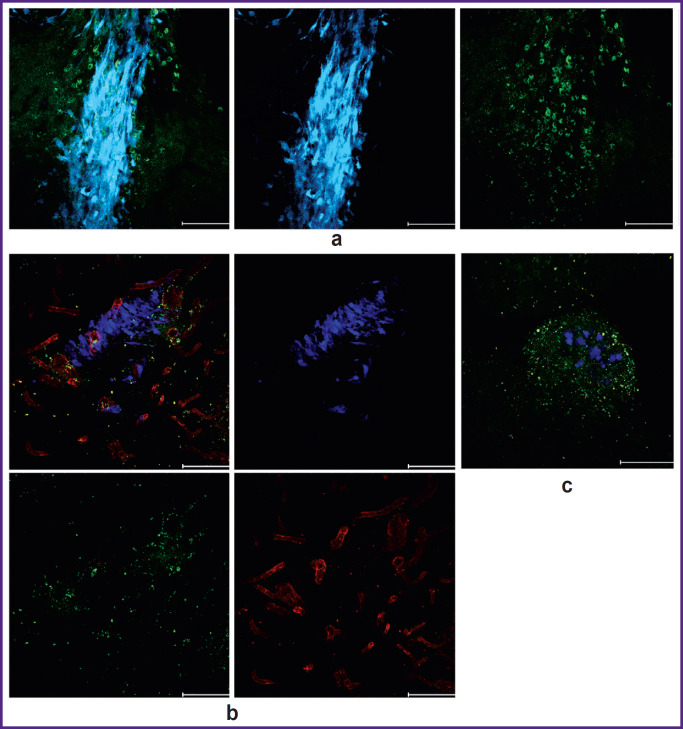
Confocal laser scanning microscopy of brain sections on days 9 and 16 after GL-261-BFP implantation: (a) Treg-eGFP (green channel) in the GL-261-BFP glioma focus (blue channel); (b) glioma invasive growth focus on day 16, the section is stained with antibodies against CD105^+^ (red channel); tumor neovascularization due to CD105^+^ cells is visualized; (c) the area of GL-261-BFP cell invasion surrounded by eGFP^+^/FoxP3^+^ Treg. Bar — 100 μm

Fluorescence imaging of tumor sections revealed that eGFP^+^/FoxP3^+^ Tregs accounted for about 50% of the total number of immune cells in the tumor mass and peritumor space on days 9 and 16 (Figure 5 (a)). Staining with antibodies against CD105 revealed intensive tumor neoangiogenesis (Figure 5 (b)). Remarkably, eGFP^+^/ FoxP3^+^ Tregs surrounded all areas of glioma invasion detected by us (Figure 5 (c)).

### Flow cytometry

**Figure 6.**
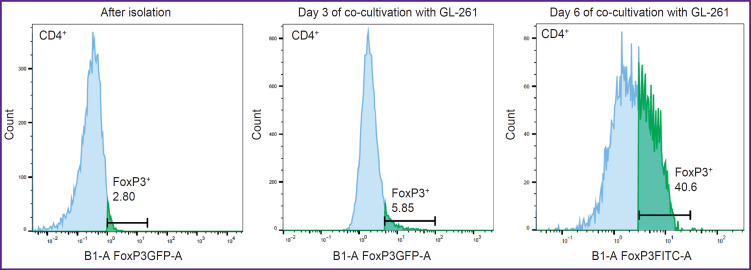
Proportion of CD4^+^ FoxP3^+^ Treg in the isolated CD4^+^ tumor-infiltrating lymphocytes immediately after isolation from the tumor, on days 3 and 14 after co-cultivation with GL-261 glioma cells

Scanning confocal microscopy is just a semi-quantitative method, therefore, to verify the content of Tregs in the tumor and peritumor space, we harvested the tumor on day 6 after implantation, suspended the cells, and estimated the proportion of Tregs from the total number of CD4^+^ T cells in the obtained TILs by flow cytometry. This study has confirmed that at early stages of tumor growth, the proportion of Tregs in the tumor microenvironment does not exceed 5% (in our experiment, the proportion of Tregs was 2.8% immediately after tumor isolation). To prove the phenomenon of increasing Tregs fraction during glioma development revealed by IVM and fluorescence microscopy, we undertook an *in vitro* experiment of culturing the obtained TILs on the feeder layer of GL-261 glioma cells. It was found that on day 3 of co-culture with glioma cells, the share of Tregs was already 5.8%, and on day 14 the proportion of Tregs was 40.6% (Figure 6). At the same time, in the control, when suspension TILs were separated from adhesive glioma cells, the proportion of Tregs did not change during the co-culture. The experiment was repeated three times with tumors from different mice and the results were reproducible: the Treg fraction increased by more than 10 times as the result of co-culturing with GL-261.

Thus, our *in vivo* and *in vitro* studies allowed us to evaluate the dynamics of Tregs recruitment by GL-261 intracerebral glioma and demonstrate selective expansion of Tregs in TILs culture, which was probably induced by cytokines secreted by glioma cells. Furthermore, we have found that Treg-eGFP isolated from the transgenic mouse tumor can be maintained in the culture mimicking the tumor microenvironment *in vitro*.

## Discussion

Currently, many aspects of functioning of the multicomponent cellular microenvironment of glioblastoma remain poorly understood. The mechanisms of recruitment and expansion of various subpopulations of immune cells, especially Tregs, are yet to be investigated and clarified in the context of immunosuppression.

The role of Tregs in immunosuppression in malignant neoplasms is well known. The transcription factor FoxP3 is a key player in the activation and function of Tregs. Since this protein has intracellular localization, routine immunochemical detection of FoxP3 Tregs using specific antibodies requires cell fixation and permeabilization, which makes intravital studies impossible. In this regard, the transgenic C57Bl/6-FoxP3-eGFP mouse line, in which FoxP3 expression is accompanied by eGFP fluorescence, appears to be an ideal platform for the intravital study of Treg activation and inactivation mechanisms during tumor development, including the intravital microscopy method.

**Figure 7.**
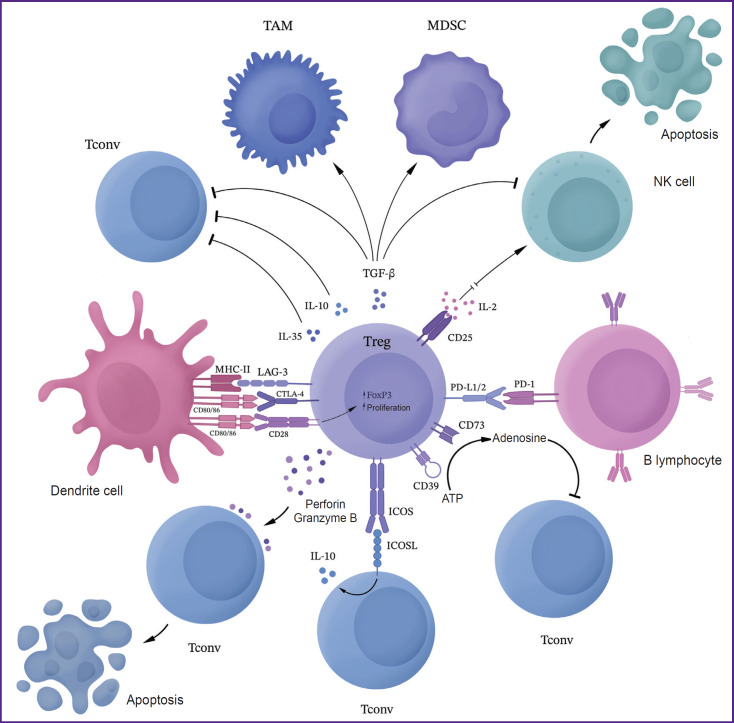
Mechanisms of the immunosuppressive action of regulatory Tregs in the tumor microenvironment Tregs — regulatory T lymphocytes; Tconv — conventional T lymphocytes; TAM — tumor-associated macrophages; MDSС — myeloid-derived suppressor cells; ICOS — membrane protein, inducible T cell costimulator; LAG-3 — membrane protein, product of the lymphocyte activation gene-3; CTLA-4 — cytotoxic T lymphocyte-associated protein 4; PD-1 — programmed cell death protein 1; PD-L1/2 — programmed cell death receptor ligand; MHC-II — major histocompatibility complex class II; TGF-β — transforming growth factor β; ATP — аdenosine triphosphate

Gliomas enhance immunosuppression through selective chemokine-mediated recruitment of Tregs to the tumor site. It has been reported that Tregs in patients with glioblastomas have significantly higher levels of CCL2-CCR4 chemokine pathway expression compared to Tregs in healthy individuals [18]. Tregs ensure glioblastoma escape primarily by inhibiting CD8^+^ cytotoxic lymphocytes, secreting various soluble factors such as IL-10 and TGF-β [19]. Active glioma Tregs can bind to CD80/CD86 via CTLA-4, suppressing T cell activity. This Treg-activating pathway can be interrupted using anti-CTLA-4 monoclonal antibodies. However, a CTLA-4 independent pathway of Treg activation in tumors has recently been described, in which activation of the CD28 co-stimulatory signaling pathway leads to selective Treg proliferation despite CTLA-4 blockade [20]. In addition to these mechanisms, FoxP3 can induce HO-1 expression, which results in the suppression of the effector T lymphocyte proliferation. In gliomas, Tregs can inhibit dendritic cells, antigen presenting cells and other lymphocytes inhibiting the secretion of IL-2 and IFN-γ and promoting the secretion of TGF-β and indoleamine-2,3-dioxygenase (IDO), thus maintaining the immunosuppressive microenvironment [19] (Figure 7).

In our study, we have demonstrated an increase in the proportion of the Treg subpopulation in the process of orthotopic glioma development using IVM and fluorescent analysis on brain sections and have confirmed that glioma can not only recruit Tregs from immunopoietic organs, but also contribute to the selective expansion of Tregs (as demonstrated in the *in vitro* experiment using co-culture of TILs with glioma cells). The contribution of recruitment, migration and selective proliferation to the increase of Treg proportion in the peritumoral space remains to be determined; however, taking into account our experimental data, the Treg expansion is likely to occur under the influence of TGF-β and other cytokines produced by glioblastoma cells.

Using primary labeled anti-CD105-Alexa-Fluor-647, we have also demonstrated active tumor neoangiogenesis in presence of infiltration by stromal cells and Tregs. CD105 is normally expressed on mesenchymal stromal cells, and when designing this experiment, we expected to visualize tumor-associated mesenchymal stromal cells and examine their co-localization with Tregs using this approach. However, using antibodies to CD105 in our experiment, microvessels were visualized in the peritumoral space (see Figure 5), which confirms the previously reported assumption that this protein in the tumor microenvironment can be a marker of neoangiogenesis [21]

In this work, we have developed the methodological approach consisting in glioma modelling and performing IVM in transgenic C57Bl/6-FoxP3-eGFP mice, which can be used in future studies to find the ways to overcome the immunosuppressive tumor microenvironment, including suppression of Tregs activity and conversion of glioma from immunologically “cold” to “hot” state, a condition in which anti-tumor immune response in glioblastoma will become more effective.

## Conclusion

The dynamics of GL-261 glioma microenvironment infiltration by regulatory T cells has been investigated. Glioblastoma cells were demonstrated to activate Tregs in the peritumor region *in vivo* and promote their selective expansion when co-cultured with TILs *in vitro*. These data can be used for further investigations on C57Bl/6-FoxP3-eGFP mice to find approaches to overcome immunosuppressive tumor microenvironment and inactivate Tregs in glioblastoma.
